# Thermoelectric Properties of NiCl_3_ Monolayer: A First-Principles-Based Transport Study

**DOI:** 10.3390/nano10030411

**Published:** 2020-02-27

**Authors:** Jing Liu, Xiaorui Chen, Yuhong Huang, Hongkuan Yuan, Hong Chen

**Affiliations:** School of Physical Science and Technology, Southwest University, Chongqing 400715, China; jingliu2112@163.com (J.L.); chenxr0909@163.com (X.C.); huangyh211@163.com (Y.H.); yhk10@swu.edu.cn (H.Y.)

**Keywords:** two-dimensional transition metal halide, spin-gapless semiconductor, thermoelectrics, power factor, lattice thermal conductivity, figure of merit

## Abstract

By employing the first-principles-based transport theory, we investigate the thermoelectric performance based on the structural and electronic properties of NiCl${}_{3}$ monolayer. The NiCl${}_{3}$ monolayer is confirmed to be a stable Dirac spin gapless semiconductor with the linear energy dispersion having almost massless carrier, high carrier mobility and fully spin-polarization. Further, NiCl${}_{3}$ monolayer processes the optimum power factor of 4.97 mWm${}^{-1}$ K${}^{-2}$, the lattice thermal conductivity of 1.89 Wm${}^{-1}$ K${}^{-1}$, and the dimensionless figure of merit of 0.44 at room temperature under reasonable carrier concentration, indicating that NiCl${}_{3}$ monolayer may be a potential matrix for promising thermoelectrics.

## 1. Introduction

Recently, two-dimensional (2D) transition metal trihalides have stimulated a large number of interest due to the low dimensional magnetism. These compounds in their bulk phase are known to exist in the form of layered materials bonded to one another through weak van der Waals interactions [1]. Generally, all of the layered compounds have been reported to adopt either the monoclinic AlCl${}_{3}$-type structure or the rhombohedral BiI${}_{3}$-type structure. They can be easily exfoliated into atomically thin 2D materials from their bulk phases due to the weak interlayer van der Waals interactions. Several transition metal halide monolayers have been successfully prepared experimentally and their magnetism have been reported [2,3,4]. Some transition metal halide monolayers have been theoretically predicted to have ferromagnetic or anti-ferromagnetic ground states [5,6,7,8,9,10,11,12,13,14,15,16,17]. Among them, Ni-bases trihalides is theoretically predicted to be Dirac spin-gapless semiconductor with the Curie temperature above room temperatures [10,16,18]. However, relevant works about lattice thermal conductivity and thermoelectric (TE) properties of these monolayers are still lacking to date.

Generally, the thermally-driven electrical performance of TE materials is measured by the power factor $PF=S^{2}\sigma$, in which *S* and σ are respectively Seebeck coefficient and electrical conductivity, while a high heat-to-electricity conversion efficiency is scaled by the dimensionless figure of merit $zT=S^{2}\sigma T/\left(\kappa_{e}+\kappa_{l}\right)$ [19,20], where *T* is the absolute temperature, $\kappa_{e}$ and $\kappa_{l}$ are electronic and lattice thermal conductivity, respectively. Obviously, the prerequisite for thermoelectrics to efficiently convert unavoidable waste heat is to search for optimum zT materials to possess a maximum PF and simultaneously a minimum κ. Unfortunately, *S* and σ unusually interweave and behave in an opposite trend [21], and σ is proportional to $\kappa_{e}$ as governed by Wiedemann-Franz’s law. Thus, the improvement of thermoelectric devices strongly depends on the optimization of electronic and thermal transport properties of TE materials. Fortunately, the 2D materials could alleviate the coupling between *S* and σ due to the quantum confinement effects [22,23], consequently enhancing thermoelectric performance [24,25,26].

Typically, the 2D NiCl${}_{3}$ monolayer is predicted theoretically to be robust ferromagnetic Dirac spin-gapless semiconductor and process the Curie temperature above the room temperature [10,16]. Its Fermi velocity ($v_{F}$) is $4.00\times 10^{5}$ m/s, approaching half that of natural graphene ($8.50\times 10^{5}$ m/s). The energetically, dynamically and mechanically stability of the material was further confirmed by its Young’s modulus calculation and *ab initio* molecular dynamics (AIMD) simulations. The zero gap between Dirac cones will be opened in NiCl${}_{3}$ monolayer with consideration of spin-orbital coupling (SOC) [10]. Thus, NiCl${}_{3}$ monolayer can also be transformed to be a Chern insulator. The unique band structure of NiCl${}_{3}$ monolayer enable it to be promising for developing efficient thermoelectrics. In this work, we evaluate the lattice thermal conductivity and thermoelectric properties of NiCl${}_{3}$ monolayer by using first-principles in combination with Boltzmann transport theory.

## 2. Computational Process

Firstly, we determine the stable configuration and the corresponding energy band structure of NiCl${}_{3}$ monolayer by employing the density functional theory (DFT) as implemented by the Vienna Ab-initio simulation package (VASP) [27,28,29] with projector-augmented wave (PAW) method [30]. The Perdew-Burke-Ernzerhof (PBE) [31] manner with generalized gradient approximation (GGA) is selected as the exchange-correlation functional. After a strict convergence test, a plane-wave kinetic energy cutoff of 500 eV is adopted and 15 × 15 × 1 *k*-mesh is chosen as the Monkhorst-Pack uniform *k*-point sampling [32] in the Brillouin zone. The structure is fully relaxed until the energy difference between two successive ionic relaxation steps is less than 10${}^{-6}$ eV and the forces on each atom are less than 0.01 eV/Å. Perpendicular to the monolayers, a vacuum of 15 Å is applied to prevent the spurious interactions between the adjacent NiCl${}_{3}$ monolayers among replica images.

Secondly, having the information of the band structure, we estimate the electronic transport properties of the NiCl${}_{3}$ monolayer, including *S*, σ, and $\kappa_{e}$ by applying the semi-classical Boltzmann transport theory within the relaxation time approximation as implemented in the BoltzTrap code [33]. Since the σ and $\kappa_{e}$ are proportional to the relaxation time (τ), it is of great importance to accurately obtain τ. As a matter of fact, τ in materials is a function of temperature and carrier concentration. So far experimental measurement is the only effective route to obtain τ [34,35]. Many earlier theoretical calculations on σ and $\kappa_{e}$ were performed by adopting a constant relaxation time approximation [36,37,38], which is generally overestimated [39,40,41]. In this work, the relaxation time is evaluated by adopting the deformation potential (DP) theory [42], which was widely used to calculate the relaxation time for two-dimensional systems [43,44,45,46,47].

Finally, the lattice thermal conductivity ($\kappa_{l}$) can be obtained by solving the phonon Boltzmann transformation related to the harmonic and anharmonic interatomic force constants as performed by the ShengBTE code [48,49,50], which takes harmonic second-order interatomic force constants (2nd IFCS) and anharmonic thirdorder IFCs (3rd IFCS) as inputs. The second-order IFCs of monolayer NiCl${}_{3}$ were computed using finite-difference method by the Phonopy code [51] using a 4 × 4 × 4 supercell with 5 × 5 × 1 *k*-point sampling. The anharmonic 3rd IFCS are obtained by Thirdorder code using a 4 × 4 × 4 supercell and Γ-point only calculations.

## 3. Results and Discussion

### 3.1. Crystal and Electronic Structures

**Figure 1:**
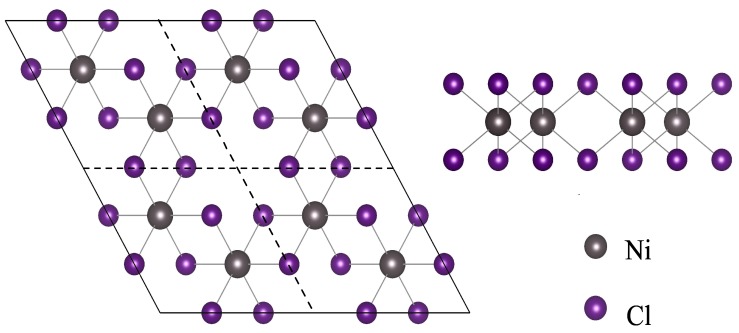
The top and side view of NiCl${}_{3}$ monolayer, respectively.

Two-dimensional NiCl${}_{3}$ layer has a hexagonal crystal structure with the No. 162 space group. Ni atoms are arranged in a two-dimensional hexagonal honeycomb shape. The six nearest Cl atoms around the Ni atom form an octahedron. Each Ni atom is the center of the octahedron, and Cl atoms are located at the six vertices of the octahedron. The obtained stable structure of 2D NiCl${}_{3}$ with the lowest energy by fully relaxing the atomic position is shown in Figure 1. After considering the DFT-D3 dispersion correction method, the Ni-Cl bond length is 2.30 Å and a lattice parameter is 5.96 Å, which is consistent with the previous theoretical work [10]. The calculated value of magnetic moment per Ni atom is 0.94 $\mu_{B}$. The total magnetic moment is 2 $\mu_{B}$ per unit cell. The ferromagnetic ground state can be identified by non-zero total magnetic moment per unit cell, which originates primarily from Ni atom. Based on calculated value of magnetic moment, Ni${}^{3+}$ is found to exist in low spin state (1 $\mu_{B}$) [16] due to high crystal field splitting.

**Figure 2:**
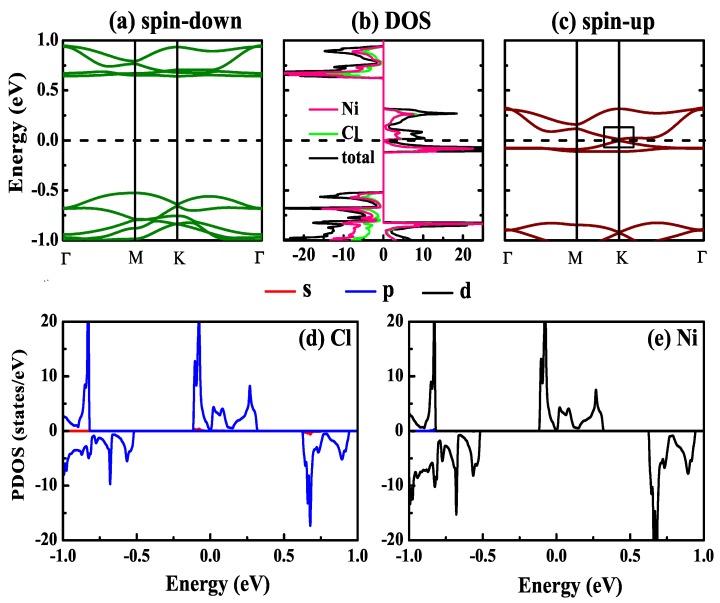
(**a**,**c**) are the calculated band structure for spin-down and spin-up channels of NiCl${}_{3}$ monolayer, respectively. (**b**) is the total DOS and atom-resolved local DOS. (**d**,**e**) are the project DOS for the Ni and Cl atoms.

The electronic properties of NiCl${}_{3}$ monolayer are characterized by the electronic density of states (DOS) and electronic band structure, which are presented in Figure 2. Obviously, there is a bandgap of 1.17 eV and the Fermi level is in the gap for spin-down channel in the Figure 2a. The spin-up band is shown in Figure 2c. The valence and conduction band at Fermi level show linear energy dispersions with zero gap between conduction band minimum (CBM) and valence band maximum (VBM) at the high-symmetry K point, indicting Dirac spin-gapless states. Besides, the DOS in Figure 2b also shows that the spin-up state does not cross the Fermi level but has a zero band gap, while spin-down states has a 1.17 eV bandgap. Band edges of both spin channels consist of the *d*-orbital of the Ni atom and the *p*-orbital of the Cl atom, as shown by the projected DOS (PDOS) in the Figure 2d,e for Ni and Cl, respectively. As a result, NiCl${}_{3}$ monolayer belongs to a Dirac spin-gapless semiconductor.

The effective mass ($m_{ij}^{*}$) in the vicinity of Fermi level, a crucial parameter for the transport behavior of semiconductor, can be extracted from using the existing band structure. Generally, $m_{ij}^{*}$ can be obtained by $m_{ij}^{*}=\hbar^{2}{\left[\frac{\partial^{2}E}{\partial k_{i}\partial k_{j}}\right]}^{-1}$, where *ℏ* is Plank’s constant, *i* and *j* represent Cartesian coordinates. Based on the the VBM and CBM, the obtained values of $m^{*}$ for the electron and hole carriers in both spin channels are listed in Table 1. By comparison, it can be found that the effective mass of the two carriers in the spin-up channel is much smaller than those in the spin-down channel, which is attributed to the linear energy dispersion in spin-up channel. In the spin-up channel, the effective mass of electrons ($m_{e}^{*}$ = 0.28 m${}_{e}$) is greater than that of holes ($m_{h}^{*}$ = 0.23 m${}_{e}$) due to the CBM is smoother than VBM, to which it is exactly opposite in the spin-down channel.

**Figure 3:**
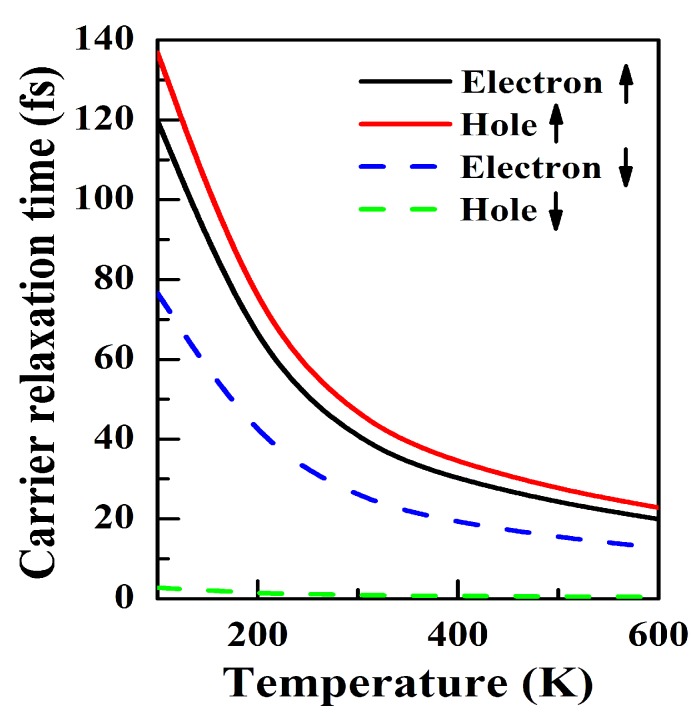
The obtained relaxation time for electron and hole for both spin channels when the temperature enhance from 100 K. ↑ (↓) represents spin-up (spin-down) direction.

The carrier mobility (μ) is also a parameter to characterize the transport ability of carriers of NiCl${}_{3}$ monolayer. Based on the Bardeen-Shockley deformation potential (DP) theory in two-dimensional materials, the carrier mobility in 2D lattice can be evaluated by [43,44,45,46,47]

(1) $$\mu =\frac{e\hbar^{3}C_{2D}}{k_{B}Tm^{*}m_{d}E_{l}^{2}},$$

where *T* is the thermodynamic temperature and $k_{B}$ represents the Boltzmann constant. $m_{d}$ denotes the average effective mass obtained by $m_{d}=\sqrt{m_{x}^{*}m_{y}^{*}}$ with $m_{x}^{*}$ and $m_{y}^{*}$ being effective mass along the *x* and *y* paths, respectively. $E_{l}$ is the DP constant determined by

(2) $$E_{l}=\frac{\partial E_{edge}}{\partial (l/l_{0})},$$

where $E_{edge}$, *l* and $l_{0}$ are the energy of CBM (or VBM), the deformation and equilibrium lattice constants, respectively. $C_{2D}$ indicates the elastic constant of 2D structure in form of

(3) $$C_{2D}=\frac{1}{S_{0}}\frac{\partial^{2}E}{\partial {\left(l/l_{0}\right)}^{2}},$$

where $S_{0}$ and *E* are the equilibrium cell area and the total energy for NiCl${}_{3}$ monolayer. For the spin-up channel, since the CMB and VBM are in contact at point K, the values of $E_{edge}$ at the CBM and VBM are exactly same, thus resulting an identical $E_{l}$ of 7.52 eV. However, there are different $E_{l}$ for holes ($E_{l}$ = 7.76 eV) and electron ($E_{l}$ = 3.53 eV) in the spin-down channel. The elastic modulus of NiCl${}_{3}$ monolayer is 154.5 Nm${}^{-1}$. Thus in the spin-up channel, the obtained mobilities of holes and electrons are $\mu_{h}$ = 1.39 × 10${}^{3}$ and $\mu_{e}$ = 1.01 × 10${}^{3}$ cm${}^{2}$ V${}^{-1}$ s${}^{-1}$). The relaxation time τ of the carriers can then be estimated by $\tau =\mu m^{*}/e.$ The resulting dependence of τ on *T* is shown in Figure 3. Obviously, τ decreases as the temperature increases and the degree of decline gradually decreases. All the needed parameters ($m_{d}$, $E_{l}$, $C_{2d}$, μ and τ) at room temperature are summarized in Table 1.

### 3.2. Thermoelectric Properties

In order to explore the thermoelectric performance of NiCl${}_{3}$ monolayer, we calculate thermoelectric transport parameters including *S*, σ and κ. Since the Curie temperature of NiCl${}_{3}$ monolayer was predicted to be about 400 K by mean-field theory [10], we only take three typical temperatures (300, 350 and 400 K) in the whole calculations.

**Figure 4:**
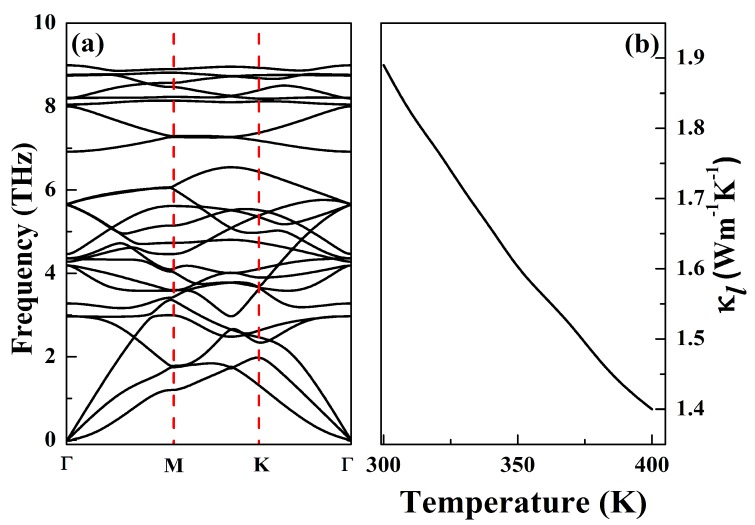
Phonon dispersion spectra (**a**) and lattice thermal conductivity (**b**) of NiCl${}_{3}$ monolayer.

Firstly, we would like to examine the phonon transport properties of NiCl${}_{3}$ monolayer, as the thermal transport in semiconductors is dominated by the phonons. The phonon spectra dispersions of NiCl${}_{3}$ monolayer are shown in Figure 4a. Since each unit cell of NiCl${}_{3}$ monolayer consists of eight atoms, its corresponding phonon dispersion has three acoustic phonon branches (ZA, TA and LA) and twenty-one optical phonon branches. The bottom three lines are acoustic phonon branches, and the others are optical branches branches. Due to the limited size of the supercell, the received phonon spectrum possesses minimal imaginary frequencies of 0.017 THz at the Γ point. Nevertheless, the structural stability of NiCl${}_{3}$ monolayer is not affected by the size of supercell [10,52]. The dispersion of all the three acoustic modes is quite stronger away from the zone center, thus giving higher velocity modes than typical optical phonon and possess most of the heat. Moreover, there is no phonon gap between acoustic and optical branches, thus indicating a strong optical-acoustic phonon scattering which will suppress the $\kappa_{l}$. Figure 4b shows $\kappa_{l}$ from 300 to 400 K. Obviously, the higher the temperature, the more intense the phonon scattering, so the $\kappa_{l}$ decreases with increasing temperature. The value of $\kappa_{l}$ at room temperature is 1.89 Wm${}^{-1}$ K${}^{-1}$. Such a low $\kappa_{l}$ implies that NiCl${}_{3}$ monolayer could have favorable thermoelectric performance.

**Figure 5:**
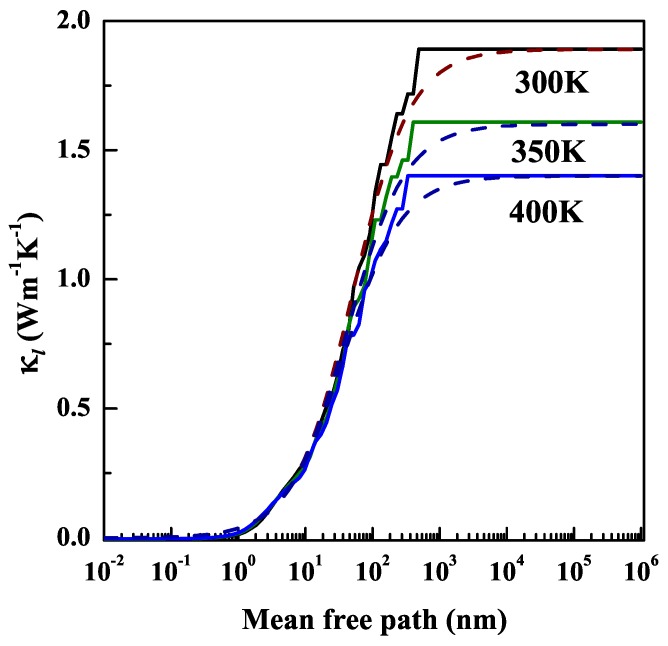
The obtained lattice thermal conductivities as a function of MFP at different temperatures. The fitted curves are indicated by dotted lines.

All samples have finite size in practical experiment and device application, thus the additional boundary scattering will reduce $\kappa_{l}$ at nanoscale or at low temperatures. Here, the size effect is estimated from the $\kappa_{l}$ as a function of the phonon mean free path (MFP). The computed κ${}_{l}$ as a function of phonon MFP at the temperature of 300, 350 and 400 K is shown in Figure 5. It can be seen that the range of phonon MFP that has significant effect on $\kappa_{l}$ is approximately 10 to 150 nm. The characterized phonon MFP denoted by Λ can be obtained by fitting the acquired $\kappa_{l}$ using a single parameter function [49]:

(4) $$\kappa_{l}\left(\Lambda \leq \Lambda_{max}\right)=\frac{\kappa_{lmax}}{1+\frac{\Lambda }{\Lambda_{max}}},$$

where $\kappa_{lmax}$ is κ of infinite size and $\Lambda_{max}$ is maximal MFP. The Λ is 50.29, 41.79, and 36.97 nm at the temperature of 300 K, 350 K, and 400 K, respectively. This indicates that the κ${}_{l}$ of NiCl${}_{3}$ monolayer may be drastically reduced when the sample size is several tens of nm. This feature is more conducive to the electronic and thermoelectric material based on NiCl${}_{3}$ in low dimension.

**Figure 6:**
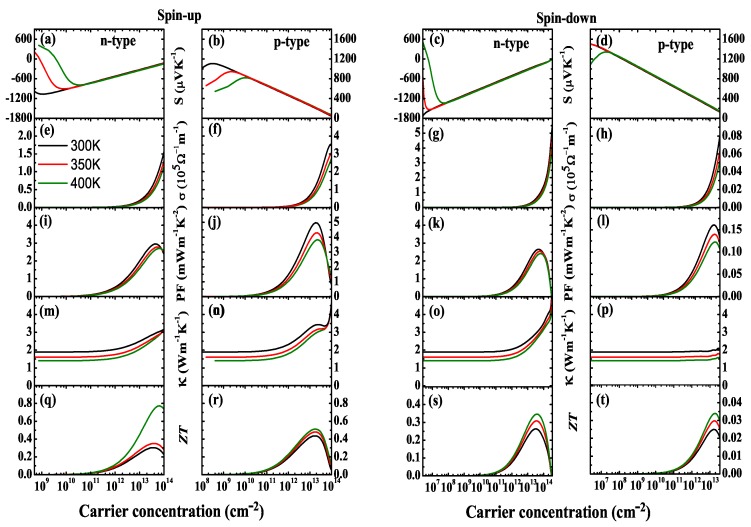
Seebeck coefficient S (**a**–**d**), electrical conductivity σ (**e**–**h**), power factor (PF) (**i**–**l**), total thermal conductivity (**m**–**p**) and zT value (**q**–**t**) of NiCl${}_{3}$ monolayer for n-type and p-type in both directions as a function of the carrier concentration at the temperature of 300 K, 350 K, and 400 K.

Secondly, the *S*, σ and $\kappa_{e}$ can be evaluated based on the band structure. Figure 6a–d show the obtained Seebeck coefficient *S* in *n*- and *p*-type NiCl${}_{3}$ monolayer as a function of carrier concentration (*n*) at *T* = 300, 350 and 400 K for the spin-up and spin-down channels, respectively. In the case of low carrier concentration, *S* is sensitive to temperature; the absolute value of *S* (|S|) is larger at low temperatures such as 300 K. When the carrier concentration is above 1.0 × 10${}^{10}$ cm${}^{-2}$, *S* is no longer dependent on temperature, but the |S| decreases as the carrier concentration increases. The values of *S* for electrons and holes are almost identical in both spin channel. For example, at the carrier concentration of 1.15 × 10${}^{9}$ cm${}^{-2}$, the |S| is 1067 μVK${}^{-1}$ for the electron at the room temperature in the spin-up channel, which is comparable to 1048 μVK${}^{-1}$ for the hole. However, the |S| in the spin-up channel is lower than the spin-down channel, although the spin-down channel exists a band gap. This indicates that the |S| of NiCl${}_{3}$ monolayer is determined by the band edge structure rather than the band gap.

The next four figures in Figure 6e–h show the dependence of electronic conductivity (σ) on carrier concentration at different temperatures. The σ increases significantly with increasing carrier concentration, but it is not sensitive to temperature. In the spin-up channel, the σ of the hole is larger than the electron under the same condition. Due to the relation $\sigma =ne\mu =ne2\tau /m_{d}^{*}$, the effective mass and relaxation time together determine the σ at a defined carrier concentration. *P*-type carriers possess smaller $m^{*}$ and larger τ simultaneously, resulting in the greater mobility of carriers, so the σ is larger than that of n-type ones in this channel. In spin-down channel, the σ of the hole is lower than the electron, which can be explained by the data in Table 1. The two factors of PF ($S^{2}\sigma$), *S* and σ, show a opposite trend with increasing carrier concentration, which complicates the dependence of PF on carrier concentration. The calculated PF are presented in Figure 6i–l. It is obvious that the value of PF firstly increases to a maximum value and then slowly decreases with the further increases in carrier concentration. The maximal PF decreases slightly with increasing temperature. In particular, the PF of *p*-type is greater than that of the n-type carrier for the spin-up channel, because the n-type carrier has a larger σ and nearly similar *S* compared with the p-type one. This also predicts that the *p*-type NiCl${}_{3}$ monolayer may has a higher zT value.

In order to obtain the final value of zT, we explored the total thermal conductivity (κ), which is shown in Figure 6m–p. The κ is mainly contributed by the $\kappa_{l}$, so it is not sensitive to the increase in carrier concentration. The temperature has a large effect on κ, and increasing temperature leads to a decrease in κ. Based on the above discussions, we can estimated the zT value of NiCl${}_{3}$ monolayer as a function of carrier concentration at three different temperatures (300, 350 and 400 K), which is exhibited in the last four figures in Figure 6q–t. In the spin-up channel, the maximal zT value is 0.3 (300 K), 0.35 (350 K), and 0.77 (400 K) for the *n*-type system, and the results are 0.44 (300 K), 0.48 (350 K), and 0.51 (400 K) for *p*-type one. Our calculations demonstrate that the NiCl${}_{3}$ monolayer has a relatively high zT value and may be a promising thermoelectric material.

## 4. Conclusions

To summarize, we have investigated the thermoelectric performance as well as the structural and electronic properties of NiCl${}_{3}$ monolayer by employing the ab initio calculations and semi-classical Boltzmann transport theory. Firstly, the the most stable crystal structure with a lattice constant of 5.96 Å and the dynamical stability are confirmed by the geometric optimization and phonon spectrum of NiCl${}_{3}$ monolayer. Then the band structure indicates that NiCl${}_{3}$ monolayer belongs to Dirac spin gapless semiconductor having almost massless carrier, high carrier mobility and fully spin-polarization. Based on the band structure of NiCl${}_{3}$ monolayer, we estimate the effective mass, the carrier mobility and the relaxation time of the carriers by using deformation potential theory. Further, the thermoelectric parameters including seebeck coefficient, electric conductivity and electronic thermal conductivity are obtained by solving the phonon Boltzmann transport theory and the lattice thermal conductivity is evaluated by using phonon Boltzman transformation related to the harmonic and anharmonic interatomic force constants, and finally, the dimensionless figure of merit are evaluated according to the obtained thermoelectric parameters and the lattice thermal conductivity. As a result, NiCl${}_{3}$ monolayer processes the optimum power factor of 4.97 mWm${}^{-1}$ K${}^{-2}$, the lattice thermal conductivity of 1.89 Wm${}^{-1}$ K${}^{-1}$ and the dimensionless figure of merit of 0.44 at room temperature under reasonable carrier concentration. These features indicate that NiCl${}_{3}$ monolayer may be a potential matrix for promising thermoelectrics.

## Figures and Tables

**Table 1:** The calculated effective mass ($m^{*}$), average effective mass ($m_{d}$), 2D elastic modulus ($C_{2D}$), DP constant ($E_{l}$), carrier mobility (μ), and relaxation time (τ) at the room temperature in both spin channels of NiCl${}_{3}$ monolayer.

Spin Direction	Carrier Type	$m^{*}$ (m${}_{e}$)	$m_{d}$ (m${}_{e}$)	$C_{2D}$ (N/m)	$E_{l}$ (eV)	μ (×10${}^{3}$ cm${}^{2}$ V${}^{-1}$ s${}^{-1}$)	τ (fs)
Spin up	Electron	0.28	0.21	154.5	7.52	1.01	159.9
	Hole	0.23	0.18	154.5	7.52	1.39	182.7
Spin down	Electron	0.95	1.48	154.5	3.53	0.19	102.0
	Hole	6.10	8.29	154.5	7.76	0.001	3.8
